# Therapeutic Role of Green Tea Polyphenols in Improving Fertility: A Review

**DOI:** 10.3390/nu10070834

**Published:** 2018-06-27

**Authors:** Sajid Ur Rahman, Yingying Huang, Lei Zhu, Shibin Feng, Ibrar Muhammad Khan, Jinjie Wu, Yu Li, Xichun Wang

**Affiliations:** 1 College of Animal Science and Technology, Anhui Agricultural University, 130 West Changjiang Road, Hefei 230036, China; dr_sajid226@yahoo.com (S.U.R.); hyyahhb@126.com (Y.H.); zhuleiahau@126.com (L.Z.); luyifsb@126.com (S.F.); wjj@ahau.edu.cn (J.W.); lydhy2014@ahau.edu.cn (Y.L.); 2 Anhui Provincial Laboratory of Local Livestock and Poultry Genetical Resource Conservation and Breeding, Anhui Agricultural University, 130 West Changjiang Road, Hefei 230036, China; ibrar.pesh@gmail.com

**Keywords:** oxidative stress, green tea polyphenol, antioxidant, fertility, supportive therapy

## Abstract

Sperm cells are highly sensitive to reactive oxygen species (ROS), which are produced during cellular oxidation. In normal cell biology, ROS levels increase with a decreasing antioxidant response, resulting in oxidative stress which threatens sperm biology. Oxidative stress has numerous effects, including increased apoptosis, reduced motion parameters, and reduced sperm integrity. In this regard, green tea polyphenols (GrTPs) have been reported to possess properties that may increase the quality of male and female gametes, mostly via the capability of catechins to reduce ROS production. GrTPs have antioxidant properties that improve major semen parameters, such as sperm concentration, motility, morphology, DNA damage, fertility rate, and gamete quality. These unique properties of green tea catechins could improve reproductive health and represent an important study area. This exploratory review discusses the therapeutic effects of GrTPs against infertility, their possible mechanisms of action, and recommended supportive therapy for improving fertility in humans and in animals.

## 1. Introduction

Infertility affects about 15 to 30% of couples that are trying to conceive. In approximately half of these cases, the male partner is the sole contributing factor [1,2]; therefore, infertility remains a controversial problem worldwide. Male infertility is caused by numerous anatomical abnormalities, such as seminal tube obstruction or neurological disorders, resulting in abnormal spermatogenesis which weakens the function of sperm [3,4]. Many environmental factors can cause infertility, such as nutritional deficiencies and oxidative stress caused by pesticides and industrial chemicals, tobacco smoking, excessive alcohol consumption, and heat exposure to the testes, which can damage semen quality [5,6,7]. Factors such as radiation or urinary tract infections also contribute significantly to fertility. When the production of reactive oxygen species (ROS) exceeds the body’s antioxidant capacity, oxidative stress (OS) occurs [8]. This OS initiates lipid oxidation, which damages membrane integrity and increases its permeability, leading to the inactivation of cellular enzymes, resulting in cell apoptosis and structural DNA damage and ultimately, leading to decreased fertility [9,10,11].

**Figure 1:**
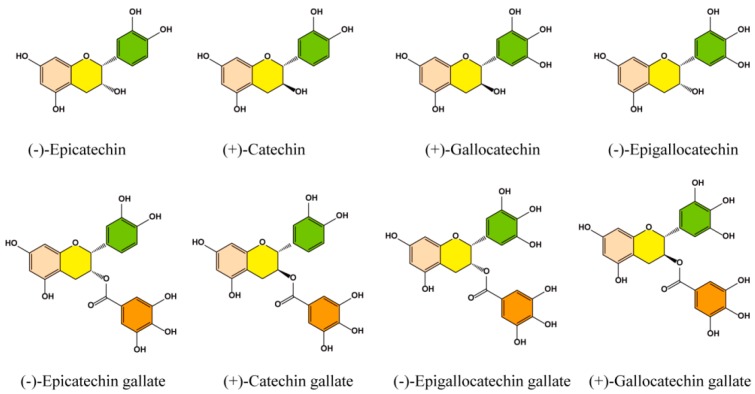
Chemical structure of the main catechin components in green tea polyphenols.

Tea is a pleasant, common, communally accepted, and safe drink that was originally used as a medicine and is now recognized as a significant industrial and pharmaceutical raw material [12]. Green tea polyphenols (GrTPs), especially epigallocatechin gallate (EGCG), have several beneficial properties, including anti-cancer [13,14,15], antioxidant, anti-diabetic, anti-hypertensive, anti-microbial, and anti-metabolic syndrome effects [16] as well as improving fertility in humans and animals [17]. Regular consumption of green tea is associated with a decreased risk of ovarian cancer in women [18]. GrTPs act as free radical scavengers, protecting spermatozoa against OS [19]. The seminal plasma comprises many enzymatic and non-enzymatic antioxidants that form a defense mechanism against the loss of semen cytoplasmic enzymes [20,21]. Studies in animals have shown that the antioxidant capacity of semen decreases under high ROS levels, and can lead to infertility-related issues [22,23]. Clinically, ROS production causes DNA damage and alters the properties of the mitochondrial membrane [24]. Green tea is considered as a dietary source of antioxidant compounds, mainly comprising polyphenolic components like catechins and gallic acid, as illustrated in Figure 1. Green tea also contains numerous other factors, such as vitamin C, carotenoids, and tocopherols; minerals, such as Cr, Mn, Se, or Zn; and certain phytochemical compounds [25]. These compounds might enhance the GrTP’s antioxidant activity [26,27]. Studies have revealed that green tea significantly increases the antioxidant capacity in plasma as well as spermatozoa after consumption of 2–6 cups/day which may lead to a decreased oxidative damage of lipids and DNA [28,29,30]. Erba and colleagues [31] proposed that green tea increases the antioxidation level and protects against oxidative damage in humans. Therefore, GrTPs regulate defensive mechanisms against oxidative damage. In this review, we discuss the possible factors responsible for infertility and the role of antioxidant activity in regulating ROS-induced infertility, and present possible solutions to increase fertility using green tea.

Recently, several studies have focused on the effects of OS, the etiology of human and animal infertility, and the role of GrTP supplements in improving semen properties to increase fertility [32]. Thus, the aim of this review was to evaluate the effects of GrTPs, their possible mechanisms in semen motility and sperm concentration, and how their use as supplements can support the treatment of infertility, through their strong capability to neutralize ROS, regulate DNA damage, and improve fertility.

## 2. Factors Affecting Fertility

Various factors affect fertility traits directly or indirectly. One of the most significant factors contributing to poor semen quality is OS [33]. Oxidative stress is a condition that induces cellular damage via ROS production [34,35]. Many in vivo experiments have been conducted to study the pathophysiology and impact of OS on different fertility disorders [36,37]. Recent research has shown that OS causes damage to semen [38], for example, through DNA structural damage and cell apoptosis, which leads to failure to conceive or arrests the development of the embryo. If the production of ROS increases in seminal plasma, OS will affect sperm motility by targeting important components of the cell, such as lipids, nucleic acids, proteins, and sugars [39,40].

The sperm membrane comprises polyunsaturated fatty acids which are highly susceptible to a chain reaction of ROS known as lipid peroxidation. Lipid peroxidation results in the loss of membrane integrity which disrupts cell function and damages sperm motility by inducing apoptosis [33,41]. The addition of metal ions, like Fe^2+^, accelerates lipid peroxidation, disrupting semen function and motility [42]. During the long-term storage of semen, lipid peroxides are freely generated in the semen membrane which induces DNA damage and reduced fertility. Lipid peroxidation also significantly reduces the capability of sperm to bind with the heterologous zona pellucida [43].

**Figure 2:**
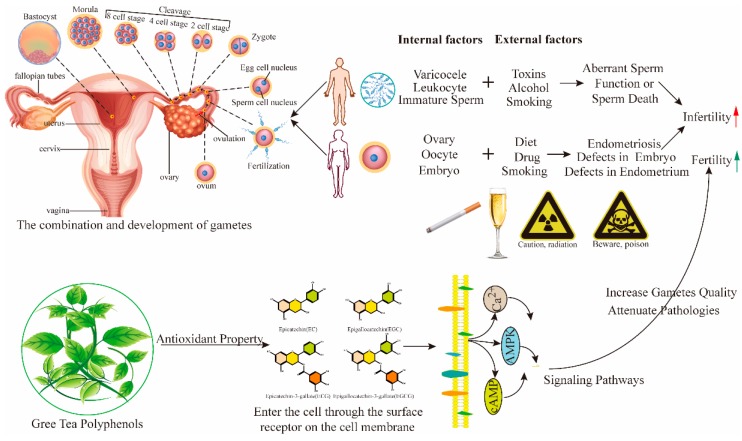
Action of green tea polyphenols on major fertility reducing factors. Various internal and external factors impair sperm function and/or cause sperm death, whereas in females, they disrupt gamete and embryo development. AMPK, adenosine monophosphate-activated protein kinase; cAMP, cyclic adenosine monophosphate; Ca^2+^, calcium ion.

Different growing cells generate ROS when incubated in aerobic conditions. ROS is a term used to describe oxygen radicals which impact on fertility and reproduction [44]. Different sources of ROS can activate leukocytes which have harmful effects on semen morphology, concentration, and motility via acrosomal damage, DNA damage, hyperactivation, and oocyte penetration [43,45]. The induction of apoptosis in antral follicles is initiated by ROS which play a vital role in primary follicle death [46]. ROS are highly reactive and oxidize other molecules that may cause structural and functional changes leading to cellular damage [47]. The different sources of ROS that impair the function of semen internally and externally are shown in Figure 2. The exogenous sources are smoking, alcohol, toxins, and radiation, while the endogenous sources are immature sperm, leukocytes, varicoceles, and cryptorchidism, as well as oocytes, embryo, ovary, and the uterine cavity [48]. These factors can alter semen parameters, induce lipid peroxidation, modify sperm proteins, and ultimately, cause sperm DNA damage, resulting in male infertility. In females these factors cause ovarian endometrium, hyperandrogenaemia, hyperinsulinemia, and placental dysfunction, which decrease fertility rate [49]. These abnormalities induce the onset of endometriosis and polycystic ovarian syndrome, causing defects in the endometrium and embryo, reducing fertility, and causing infertility [50].

## 3. Antioxidants and Infertility

Previous studies have confirmed the significant roles of antioxidants in male and female infertility and other pathological conditions [51]. Antioxidant supplementation has been specified as a possible approach to treat reproductive diseases and improve fertility. In 2017, it was proven, for the first time, that polyphenols found in significant amounts within the seminal plasma act as natural antioxidants [52].

### 3.1. Vitamin E

Several studies have reported that vitamin E is a key chain-breaking antioxidant in the seminal plasma membranes because it favors the motility of semen by resisting the peroxidation of lipids [53]. It also deactivates free radicals, protects the cellular membrane against O_2_ free radicals, and prevents the production of ROS [54]. During cryopreservation, when vitamin E reacts with cryoprotectants, it can efficiently preserve the semen [55,56,57]. Vitamins E and C collectively enhance the intra-cytoplasmic semen injection (ICSI) success rate in patients with semen DNA damage and decrease the level of DNA impairment [58]. Glutathione (GSH) has a protective effect on sperm motility. Parinaud and colleagues [59] compared GSH with Tyrode solution and observed increased recovery of sperm motility with GSH.

### 3.2. L-Carnitine

Studies have revealed that L-carnitine has an impact on animal and human infertility. Lenzi and coworkers [60] identified a positive relationship between L-carnitine, L-acetyl carnitine, and semen motility in infertile men. When patients received a placebo for more than 3 months with L-carnitine therapy (2 g/day), their sperm showed higher motility. Short-term administration of L-carnitine had a positive effect on the sperm count, leading to successful pregnancy [61]. In addition, albumin successfully avoids the proliferation of peroxidative impairment in sperm by acting as an antioxidant [62].

### 3.3. Co-Enzymes

Co-enzyme Q10 and catalase act as antioxidants that preserve and increase semen motility [63,64,65,66]. Co-enzyme Q10 is associated with semen parameters, such as concentration, morphology, and motility [6]. Thakur and colleagues [67] proposed that regular supplementation of co-enzyme Q10 at 150 mg increases sperm parameters in infertile males; however, it was not shown to improve the live birth or pregnancy ratio [68].

### 3.4. Superoxide Dismutase

Superoxide dismutase (SOD) is an important antioxidant enzyme that has a significant role in scavenging ROS, such as hydrogen peroxide (H_2_O_2_) and superoxide anion, thereby protecting sperm from peroxidation. For example, high concentrations of SOD prevent the loss of semen motility (*p* < 0.005) [69]. Negri and coworkers [70] showed that a SOD-based antioxidant supplementation, hydroxytyrosol and carnosol, improves sperm DNA integrity. SOD prevents ROS generation and provides complete protection when added with catalase (0.008 mg/mL) to semen suspensions [71]. Catalase and SOD also have positive effects against sperm intracellular (mitochondrial and plasma membrane) and extracellular (leukocytes) ROS, and promote sperm motility [72,73].

### 3.5. Selenium

Selenium (Se) is a vital trace element that is important for many physiological processes, such as immune and reproductive systems, the metabolism of thyroid hormones, and antioxidant protection [74]. Selenium plays a vital role in the creation of testosterone and in semen biosynthesis. Selenium and *N*-acetylcysteine treatment significantly enhances semen motility. In clinical trials [75], 30 weeks of treatment with selenium and *N*-acetylcysteine in 468 infertile males successfully decreased follicle-stimulating hormone; however, it increased the semen inhibin B and testosterone levels. Rezaeian et al. [76] suggested that 5 mg/mL selenium could improve sperm parameters and sperm vitality after freezing and thawing procedures which could be utilized in clinics to treat the infertility of sub-fertile men. Some placebo trials in different countries have confirmed that selenium administration successfully increases motility, morphology, and sperm counts in infertile males [77]. In a descriptive study conducted by Nenkove et al. [78], a high level of Fe and a low level of Se were the major causes of sperm damage. Variation in trace mineral concentrations in animals’ seminal plasma may be associated with semen quality because trace minerals are involved in the maintenance of the pro-antioxidative balance during ejaculation. It is suggested that antioxidants and micronutrients may be used to protect spermatozoa from the high production of ROS that occurs in many clinical circumstances [79].

## 4. Green Tea Polyphenols Improve Fertility

Tea is derived from the *camellia sinensis* plant in the form of green, black, and Oolong tea, a popular beverage consumed globally, which accounts for about 3 billion kilograms (kg) of annual production. Green tea is common and preferred in China, Japan, and certain other Asian countries. Green tea polyphenols (GrTPs) are potentially effective in ameliorating inflammatory bowel disease (IBD) and related disorders via their known anti-inflammatory, antioxidative, and anti-bacterial properties. GrTPs have been shown to reduce inflammatory reactions by targeting several signaling pathways [80]. GrTPs downregulate IκB kinase (IKK), c-Jun N-terminal kinase-mitogen-activated protein kinase (JNK-MAPK) [81], nuclear factor*-*Kappa B (NF-kB), cytokine-like tumor necrosis factor-α (TNF-α), cyclooxygenase-2 (Cox-2), and B-cell lymphoma (Bcl-2), to protect against hepatic disorders and numerous chronic and inflammatory disorders [82,83]. Moreover, GrTPs also act on extracellular signal-regulated kinases (ERK) and Akt signaling pathways as anti-itch therapies in other skin diseases [84,85]. Green tea contains high quantities of polyphenols which generate oxidized proteins that act as natural antibodies [86]. The antioxidant properties of polyphenols comprise scavenging and destroying free radicals. Testicular tissue is predisposed to suffer from the action of free radicals and OS because of its high cell division rate, oxygen consumption rate, and low oxygen pressure, debilitated vessels, and high levels of unsaturated fatty acids [87]. In addition to its antioxidant properties, green tea may also decrease inflammation, reduce DNA fragmentation, and increase the motility and viability of semen [88]. Other potential benefits of polyphenols include improved egg viability and reduced cellular damage of reproductive organs. In addition, its antioxidative concentration correlates with sperm levels and motility [89].

**Figure 3:**
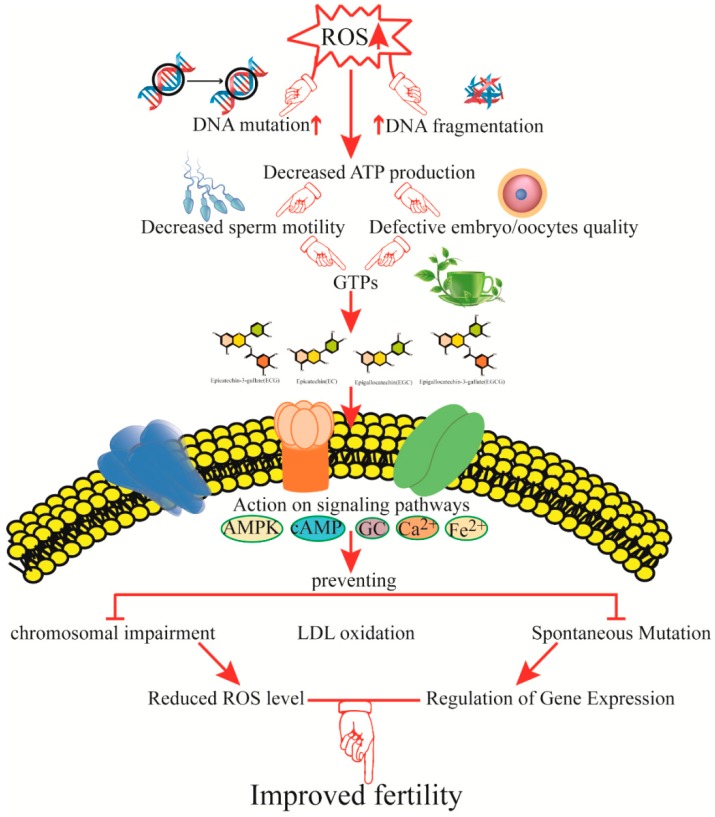
Mechanisms by which green tea catechins improve fertility and reproductive function. The figure shows potential mechanisms of action of green tea catechins in different pathways and proposes that green tea polyphenols (GrTPs) are capable of improving fertility by improving sperm and embryo quality. ROS, reactive oxygen species; ATP, adenosine triphosphate; AMPK, adenosine monophosphate-activated protein kinase; cAMP, cyclic adenosine monophosphate; Ca^2+^, calcium ion; Fe^2+^, ferric iron; LDL, low-density lipoprotein.

Normally, sperm cells produce significant amounts of ROS during their physiological metabolism. Testicular tissue comprises a high quantity of unsaturated fatty acids that are very sensitive to ROS. ROS have devastating effects via lipid peroxidation which damages the structure of the lipid matrix in semen membranes and reduces intracellular levels of adenosine triphosphate (ATP) which may lead to reduced sperm viability, axonemal injury, and an increase in mid-piece morphological defects [90]. A study in rats indicated that testicular torsion-detorsion causes OS which may lead to decrease semen concentrations in the testes [91]. GrTPs are water-soluble components, comprised of (−)-epigallocatechin gallate (EGCG), (−)-epicatechin gallate (ECG), (−)-epicatechin (EC), and (−)-epigallocatechin (EGC). EGCG prevents spontaneous mutation, low-density lipoprotein (LDL) oxidation, and chromosomal impairment induced by ROS in somatic cells [92] (Figure 3). Therefore, GrTPs may exert their antioxidative effects on spermatozoa. Roy and coworkers [93] found that supplementation of a Tris-egg yolk extender with polyphenols (0.5, 0.75, or 1 mg/mL) improved the motility and capability of semen. However, the addition of EGCG substances in cooling medium for stallion sperm storage is not useful [94]. The semen antioxidant system is made up of many components, including enzymatic, non-enzymatic, and low molecular weight molecules that provide maximum protection against ROS. An important component in green tea is the catechins, which have higher antioxidant activity than that of vitamin C. In rats, catechins were observed to reduce ROS levels, regulate gene expression, and maintain glucose levels [95,96]. Semen motility in infertile humans is affected by seminal ROS [97]. An increased level of seminal ROS may be associated with an increase in sperm DNA fragmentation [98]. Supplementation of semen storing media with GrTP extract has shown a dose-dependent increase in sperm capability, which has aided cases of idiopathic infertility.

Many previous studies have lacked randomization and placebo-controlled arms. The kind, amount, and period of antioxidant treatment has also varied significantly. Some researchers studied a few antioxidants, while others altered the duration of the therapy or used different combinations. Some common antioxidants that have been studied include vitamin C, vitamin E, selenium, zinc, glutathione, L-carnitine, and *N*-acetylcysteine [99,100]. When comparing EGCG with other components, very low (2 μM and 20 μM) concentrations could improve sperm motility and its capacitation [101]. The use of EGCG at different concentrations (10, 20, and 60 μM) significantly improved the number of semen bound to the zona pellucida (ZP) up to control levels, suggesting that GrTPs are able to decrease the damage caused by rotenone [102]. The addition of 25, 50, and 100 μM EGCG to thawed sperm for one hour did not have any effect on sperm viability and acrosome integrity but increased the in vitro penetration rate and the efficiency of fertilization [103]. GrTPs are expected to have impacts on the treatment and management of idiopathic infertility. An animal model study conducted by Awoniyi and colleagues [104] examined the modulation of ROS by GrTPs in rat semen. They found that GrTP supplementation significantly increased the sperm count and motility. GrTPs have also been shown to decrease lipid peroxidation, protein carbonylation, and DNA damage [105]. The proliferation of semen lipid peroxidation significantly reduces sperm motility [106]. Semen DNA damage is promoted by the enhanced generation of ROS [107]. If semen is treated with polyphenols (0.01 mol/L), the fertility rate significantly increases [108,109]. Adding albumin at 0.3–10% might enhance DNA protection and prevent DNA damage from defusing peroxides formed during lipid peroxidation [110].

GrTPs can protect fertility and provide some degree of protection from reproductive disorders, as shown in animal studies. Green tea extract provided significant protection to the growing fetus against the toxic effects of a high dose of Indomethacin [111]. It also prevented the parental mortality that is associated with this drug and showed ameliorative effects on all parameters associated with reproductive performance and OS. Green tea extract contains vigorous medical ingredients, representing a good source of natural antioxidants that improve fertility, as summarized in Figure 3.

## 5. Possible Combination of GrTPs with Different Extracts to Improve Fertility

Research on animal and human fertility and its interactions with diet has increased significantly in recent years. The dietary intake of folic acid has been consistently linked to a reduced incidence of infertility, lower risk of pregnancy loss, and better management of infertility [112]. The combination of different nutrients with GrTPs can successfully increase the motility, quality, and quantity of sperm as well as the chances of pregnancy. Numerous natural antioxidants related to both enzymatic and non-enzymatic groups can remove excess ROS and prevent OS.

### 5.1. Aspalathus Linearis

Rooibos extracts (*Aspalathus linearis*) in combination with GrTPs may defend against induced oxidative damage by increasing the antioxidant protective mechanisms, thereby, improving the semen quality and function [104].

### 5.2. Vitex Agnus Castus

Along with GrTPs, another recognized fertility herb is *Vitex Agnus castus* or chasteberry. This herb is believed to be useful for women with hormonal imbalances, irregular cycles, or reduced luteal stages (especially in the second half of the cycle) [113]. Vitex combined with GrTPs stimulates and balances the hormones that govern the menstrual cycle. It also helps to regulate pituitary gland functions which may help to balance the release of progesterone and estrogen hormone levels as well as enhancing fertility, representing a novel treatment to increase the pregnancy rate [114].

### 5.3. Pu-Erh

*Camellia sinensis* var. Assamica combined with Pu-erh black tea was observed to have no adverse effects in terms of reproductive and developmental toxicity at a level of 700 mg/kg/day [115]; however, its potential toxicity when administered at a high dose as a concentrated extract has not been completely investigated.

Thus, in the near future, combinations of green tea with different herbs or nutrients will be developed as novel therapies to improve the fertility rate and treat infertility. More information regarding the treatment of infertility using GrTPs will be gained from well-planned observational studies in the future.

## 6. Conclusions and Future Perspective

Polyphenols exhibit various effects, including increasing antioxidant (e.g., GSH and cysteine) levels in vital organs [116,117,118,119,120]. GrTP administration at low concentrations could reduce OS and ultimately improve fertility in humans and animals; however, they can exert the opposite effect at higher concentrations. A consensus is still required regarding the type and quantity of GrTPs required to gain the maximum effect. Although GrTPs have demonstrated utility in treating male and female infertility and alleviating OS their precise actions and mechanisms remain to be determined. At the molecular level, GrTPs may represent a complementary treatment to improve fertility and treat infertility or related diseases. It should also be determined whether polyphenols or their composite extracts have supplementary effects on fertility. Furthermore, future studies should combine GrTPs with different extracts or herbs to determine their effects on semen motility and the pregnancy rate as key outcomes.
